# Computational forecasting using Swedish data in the vaccination phase of the COVID-19 pandemic: a systematic literature review deliberating modelling relevance for public health and healthcare

**DOI:** 10.1186/s12913-026-15076-y

**Published:** 2026-07-10

**Authors:** Anna Saxne Jöud, Henrik Thorén, Armin Spreco, Torbjörn Lundh, Toomas Timpka, Philip Gerlee

**Affiliations:** 1 Department of Laboratory Medicine, Lund University, Lund, Sweden; 2 Department of Clinical Sciences Lund, Lund University, Lund, Sweden; 3 Department of Research and Development, Skåne University Hospital, Lund, Sweden; 4 Department of Philosophy, Lund University, Lund, Sweden; 5 Department of Health, Medicine and Caring Sciences, Linköping University, Linköping, Sweden; 6 Mathematical Sciences, Chalmers University of Technology and University of Gothenburg, Gothenburg, Sweden; 7 Regional Executive Office, Region Östergötland, Linköping, Sweden

**Keywords:** Computational modeling, COVID-19 pandemic, Preventive health services, Health policies, Models, Decision support, Biostatistics, Health informatics

## Abstract

**Background:**

The benefits of computational forecasting in the later phase of the COVID-19 pandemic when vaccines and clinical pharmaceutical interventions were available have seldom been assessed. We aimed to evaluate computational forecasting research applied to Swedish populations in the vaccination phase of the pandemic.

**Methods:**

A systematic search was performed on March 8, 2024 in the electronic databases PubMed, Scopus, Cochrane library, Embase, Love platform and Epistemikos. An updated version of the Risk of bias Opinion Tool (ROBOT) was used to assess the quality of evidence reported in the papers identified in the search. The articles fulfilling the quality criteria were assessed for suitability for meta-analysis. Data were extracted from the selected articles for synthesis of characteristics, and a thematic analysis was used for a qualitative synthesis of the contents.

**Results:**

Of 2034 unique publications identified in the database search, 6 articles satisfied the selection and quality criteria. Variability in the reporting of forecasting performance results was found to make a quantitative meta-analysis of forecast performance infeasible. The data synthesis showed that statistical modeling using Bayesian calibration was the most common methodological approach. No external model validation was reported, but 5/6 articles included internal model corroboration data. The primary theme resulting from the qualitative synthesis of article content was design or refinement of computational models with demonstration of model use in health service practice as a secondary theme. None of the articles referred to health service policymaking as the primary research context.

**Conclusion:**

Computational forecasting research using Swedish population data from the vaccination phase of the COVID-19 pandemic was deployed in a model design context. While methodological knowledge was developed, most of the research was not initiated to solve the public health and healthcare problems at hand. Our results indicate that the alignment between computational forecasting research and policymaking needs in the vaccination phase of pandemics can be enhanced.

**Supplementary Information:**

The online version contains supplementary material available at 10.1186/s12913-026-15076-y.

## Introduction

The public health response to the COVID-19 pandemic was initially restricted to non-pharmaceutical interventions (NPIs) [1]. Facing a shortage of reliable epidemiological data, health service policymakers needed during that phase of the pandemic reliable estimates of COVID-19 morbidity, mortality, and patient burden at different healthcare facilities to decide appropriate NPIs and for planning resource allocation. Many health service providers and policymakers turned to mathematical modelling, and in particular computational forecasting of epidemiological outcomes, as a means of decision support [2, 3]. These forecasts provide to the most likely future outcome of relevant endpoints given the historical data used for calibrating model parameters [4].

One year into the pandemic, in early 2021, the scientific knowledge of the pandemic disease had increased manyfold, covering aspects ranging from virological and immunological features of SARS-CoV-2 [5] to the epidemiological characteristics of COVID-19 [6, 7]. Consequently, the possibilities to protect populations had grown dramatically by improved knowledge about mechanisms of transmission as well as development of vaccines and effective clinical pharmaceuticals and procedures. Despite the expansion of knowledge, there were areas where policymakers lacked data when deciding on response strategies. For instance, it was unclear for how long the novel mrna-based vaccines provided immunity and to what extent they protected against newly detected SARS-CoV-2 variants of concern [8].

Sweden gained global attention during the early stages of the COVID-19 pandemic for its partial lock-down with, for instance, the elementary schools remaining open [9, 10]. The performance of computational prediction models applied to Swedish populations during the first pandemic year (2020) have previously been evaluated [11]. However, the COVID-19 pandemic was long and therefore the models changed over time when more data and new knowledge emerged. For decision-makers, having access to multiple models, might aid the decision process, but could also hinder decisions when models indicate different outcomes and when the models differ in terminology and measures of uncertainty. Therefore, the aim of this study was to evaluate corresponding models applied to Swedish data in the latter phase of the pandemic when vaccines and effective clinical procedures were available. Hence, this study focuses on computational prediction models for Sweden in the time period 2021–2022.

## Methods

We extended a previously published systematic review (PROSPERO no. CRD42021229514) (Supplement S1) that covered articles reporting computer forecasting models applied to Swedish population data in the period 1 January 2020 to 31 December 2020 [11]. The current systematic review covers articles reporting computer forecasting models applied to Swedish population data in a time period that follows directly after the previous systematic review, namely 1 January 2021 to 31 December 2022.

### Definitions

The terms ‘forecast’ and ‘prediction’ were interpreted to denote an unconditional prediction about what will happen in the future. A scenario was defined to be a virtual setting for conditional predictions, i.e. a model for predicting conditional on a set of assumptions about the future or eventualities that are currently unknown and for which information may or may not be accessed in the future [12].

### Database searches

A systematic search was performed in the electronic databases PubMed, Scopus, Cochrane library, Embase, Love platform and Epistemikos. The final search was made March 8, 2024. The systematic search was based both on keywords and MeSH terms such as: prediction, nowcast, forecast, simulation model, model, modelling, estimation, scenario, surveillance, Epidemiology, COVID-19, SARS-cov-2, swed*. The systematic search is described in a PRISMA-S protocol (see Related files).

### Inclusion criteria

Scientific articles that report epidemiological results regarding actual or scenario-based predictions of morbidity, mortality, or healthcare burden caused by COVID-19 in Sweden or parts of Sweden in 2021–2022.

### Exclusion criteria

Scientific articles in of the below listed classes were excluded:

- Non-original analyses e.g. reviews, perspective articles, editorials, protocols and guidelines.
- Duplicate studies.
- In silico studies, i.e. pure simulations based on virtual data.
- Descriptive epidemiological publications, e.g. description of case incidences and geographical distributions.
- Models that *only* examine the effect of interventions, rather than predicting risk or disease burden.
- Articles that present predictions covering a time period mainly outside of 2021–2022 (to avoid overlap with studies investigated in [11]).

### Assessment of relevance

After the initial literature search all identified articles were screened by two authors. Title and abstract were screened for relevance related to the Population and Intervention of the beforehand determined (PICO). This was followed by full text screening of the remaining articles. Then, all parts of the PICO were assessed. This screening was done by three co-authors. Reason for exclusion was documented. In both stages, disagreements were handled through consensus discussions. The literature searches and assessment of relevance were reported according to the PRISMA-S protocol (see Related files).

### Risk of bias assessment

The next step following the data extraction was the Risk of Bias assessment per model in each article (Supplement S3). Here we used an updated version of the Risk of bias Opinion Tool (ROBOT) created and used in the previous published systematic review of computational forecasting [11]. ROBOT was developed in response to the lack of assessment tools for epidemiological forecasting studies. It is based on two existing assessment tools: The Cochrane Collaboration’s tool for assessing risk of bias in randomised trials [13] and PROBAST, which is used for the assessment of diagnostic and prognostic prediction model studies [14]. While these tools contain some relevant items they are constructed for assessing clinical trials and diagnostic/prognostic disease models and not with computational forecasting studies in mind.

The ROBOT tool developed in [11] accounted for relevance and quality of data, time frame for prediction, assumptions, and how the model was generated. The assessment of assumptions included reproduction rates, latency period, incubation period, serial interval, infectious period, population immunity, and impact of interventions during the prediction period. Model validation was classified as one out of three: retrospective/internal validation, external validation, or no validation. We consider validation to be retrospective/internal if it is carried out on data from the same time frame and population and external if validated on another population and/or time frame [15].

The assessment of systematic sources of error was performed by one assessor and then discussed between all co-authors. Each sub-aspect was given a score rating in an assessment form, ROBOT, (see Supplement S3). The partial assessments were added up to a total score for each model. To qualify for further result synthesis, a total score below a heuristically defined limit value was required (ROBOT < 4).

### Data extraction

For data extraction we used a spreadsheet for retrieving data from each article to an excel sheet split by article and per item. The items included data on the authors’ country of origin, forecasting study design, type of forecasting model, method of calibration, forecasting outcome, mentioning of predictive uncertainty, performance measures, model documentation and model validation. Data extraction was done by two co-authors independently. Where there were differences between the two, discussion led to consensus.

### Meta-analysis and data synthesis

The possibilities to perform a meta-analysis of forecast performance were first assessed. The variability in the formatting of performance results did not allow such an analysis. A structured synthesis of the data extracted from the articles was then performed and presented. Thereafter, a qualitative synthesis using a thematic analysis based on a critical realist foundation was performed to coalesce the content of the articles [16]. In a reflective process, significant text units were identified, and the content sorted into categories and preliminary themes. The information charted from the articles ranged from the knowledge gap to be filled and study aims, to topics addressed in the introduction and discussion sections. In the final step, preliminary and secondary themes in the content were generated to provide a qualitative representation of the articles.

## Results

### Literature search

**Fig. 1:**
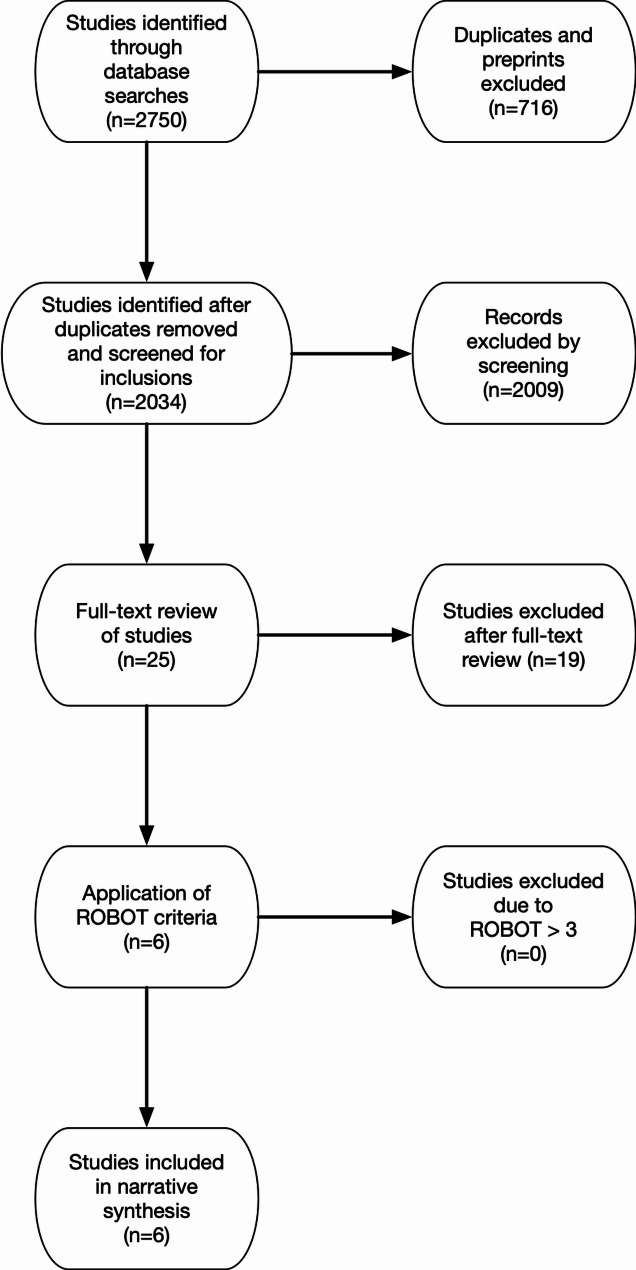
PRISMA flow-chart indicating the number of studies identified, screened, and confirmed for eligibility into this systematic review

The database search identified 2750 peer-reviewed articles published during the period 1 January 2021 to 31 December 2022 (Fig. 1). A total of 597 duplicates and 119 articles published outside the inclusion period were excluded. Hence 2034 articles remained to the first screening of title and abstracts. Here 2009 articles were excluded, leaving 25 articles for full text assessment where 19 were excluded leaving 6 to be assessed by the ROBOT. All 6 articles remained for data extraction and synthesis.

### Synthesis of extracted data

A summary of the extracted data is shown in Table 1. Four articles had at least one Swedish author (1,2,3,6) while the remaining two studies were reported by European and North American authors (4,5).

**Table 1:** Summary of data extracted from the studies included in the narrative synthesis

Study number	Title	Authors	Authors' country of origin	Forecasting study design	Type of Forecasting Model	Mention of model limitations	Method of calibration	Forecasting outcome	Mention of predictive uncertainty	Performance measure	Model Documentation	Model validation
1	Spatio-temporal predictions of COVID-19 test positivity in Uppsala County, Sweden: a comparative approach.	van Zoest et al.	Sweden	Prediction	Statistical and time-series model	Present	Least squares and Bayesian	Test positivity	Absent	MRSE	Code available	Only internal
2	Development of Forecast Models for COVID-19 Hospital Admissions using Mobile Network Data: A Privacy-Preserving Approach	Taghia et al.	Sweden	Prediction	Statistical model	Present	Not clear	Hospital admissions	Absent	MAE	Not reproducible	Only internal
3	A joint Bayesian spatiotemporal risk prediction model of COVID-19 incidence, IC admission, and death with application to Sweden.	Jaya IGNM, Folmer H, Lundberg J	Sweden, European and Asian	Prediction	Statistical model	Present	Bayesian	Incidence, ICU-admission, mortality	Absent	MAPE, MAE, RMSE, Pearson's r	Not reproducible	Only internal
4	Forecasting COVID-19 Epidemic Trends by Combining a Neural Network with R(t) Estimation.	Cinaglia P, Cannataro M	European country	Prediction	Time-series model	Absent	Not clear	Incidence	Absent	RMSE, MAPE, MAE	Not reproducible	Only internal
5	Modelling COVID-19 dynamics and potential for herd immunity by vaccination in Austria, Luxembourg and Sweden.	Kemp et al.	European country and North American country	Scenario	Compartmental model	Present	Manual and Bayesian	Daily cases, ICU occupation, Hospital occupation, Mortality	Present	None	Code available	None
6	Bayesian nowcasting with leading indicators applied to COVID-19 fatalities in Sweden	Bergström, F., Günther, F., Höhle, M., Britton, T.	Sweden	Prediction	Statistical model	Present	Bayesian	Mortality	Present	RMSE, CRPS, logS, PI-coverage	Code available	Only internal

Four articles were reported in a theoretical/academic context (3,4,5,6), whereas the remaining two publications demonstrated application of forecasts in health service practice (1,2). No external model validation was reported, but 5/6 articles in the final set included model corroboration data in some form. Estimates of uncertainty in the forecasts were reported in 2/6 articles. Statistical modeling and Bayesian calibration methods were employed in most of the articles.

Five articles (1,2,3,4,6) stated the aim of the modeling was prediction or forecasting, while one article described the aim of the modeling to be scenario development (5). In one article (1), four models were considered, three different statistical models and one time-series model. The four models were also combined into an ensemble model. The remaining articles reported three different statistical models (2,3,6), one time-series model (4), and one compartmental model (5), respectively.

The outcomes considered were positive SARS-CoV-2 test (1), hospital admissions for COVID-19 (2,5), COVID-19 incidence (3,4,5) and COVID-19 mortality (5,6). Two articles (3,5) made predictions for multiple outcomes. Four articles (1,3,5,6) reported use of a Bayesian framework for model calibration. In one of these articles (5), a manual calibration based on parameter values derived from the literature was also reported. In the article reporting an ensemble forecast by combining forecasts of several models (1), least squares estimation was used to distribute weights between the component models. Calibration data could not be extracted from two articles (2,4). A common characteristic of the modeling reported in these articles was use of neural networks.

No forecast was prospectively evaluated against the factual outcomes, but all but one article (5) reported some quantitative measure of model performance. Three articles considered more than one measure (3,4,6). Root mean squared error (RMSE) was the most common performance measure, used in four articles (1,3,4,6), followed by mean absolute error (MAE) used in three articles (2,3,4). One article (6) made use of probabilistic predictions. For quantitative evaluation of these predictions, the authors used CRPS, Log Score and PI-coverage.

Potential limitations of the modeling were mentioned in five articles (1,2,3,5,6). In one article (6), uncertainty in point predictions was displayed using prediction intervals. In the article reporting scenario models (5), uncertainty was displayed by credible intervals and reporting optimistic and pessimistic scenarios. In four articles (1,2,3,4), model uncertainty was not addressed.

Regarding model documentation, the programming code used to implement the models was in three articles (1,5,6) made available via a repository. In the remaining articles, no attempt was made to share the programming code. The models reported in these articles (2,3,4) were deemed to be non-reproducible due to the fact that no code was provided and the models were too complex to be reconstructed from the brief descriptions provided in the articles.

None of the models reported met the criterion for external validation. Five articles (1,2,3,4,6) reported some form of internal model validation, while one article did not consider any validation (5).

### Qualitative synthesis of article content

The primary theme in the final set of six articles was design or refinement of computational models with demonstration of model use in health service practice as a secondary theme. None of the articles referred to health service policymaking as the primary research aim.

A central element in the primary theme was aggregation of different data streams and algorithms to enhance point predictions. In (6), it was contended that partial delays in critical data streams may cause misleading readings of point estimates during pandemics. To correct for delays in data reporting, an extension to existing autoregression methods that incorporated regression of correlated streams of data collected upstream (on COVID-19 cases and ICU admissions) was developed to estimate the true number of fatalities on a given day. The authors demonstrated the feasibility of three variations of the method by retrospective analyses of Swedish COVID-19 mortality data showing that ICU admission data provided the best performance. Similarly aggregating data streams, (3) exploited that COVID-19 incidence, ICU admission, and mortality curves were parallel to each other, although with time lags and with unknown proportionality, for forecasting. A retrospective analysis of Swedish data carried out in (3) showed that a combined data stream model outperformed models using the individual streams, except in prediction of ICU admissions. Instead of aggregating data streams, in (1) three statistical models for one-week ahead forecasts of positive COVID-19 tests in a Swedish county were combined into an ensemble model that in a retrospective analysis was found to improve the predictive performance of the individual models.

Widening the perspective from point predictions, (5) reported a scenario model for conditional ‘what-if’ estimates of time to reach herd immunity for different vaccination rollout strategies. The authors demonstrated model feasibility using population data from several countries, including Sweden. Instead of focusing on specific issues associated with model design, (4) addressed forecasting methodology at a more general level. They postulated that COVID-19 transmission is contingent on contextual aspects such as population density, lifestyle, and vaccination coverage and that models designed for a particular geographical area risk to fit poorly with other areas. Upon these premises, the authors presented a machine learning model based on neural networks for estimation of effective reproduction numbers and demonstrate the method using Swedish data.

Regarding the secondary theme in the articles, model demonstration in health services, (1) contended that forecasts resulting from time-series analyses alone often yield wide prediction intervals, but can be useful for health service decision-making in the short-term (1–10 days). The authors therefore developed four different statistical models for one-week forecasts of positive COVID-19 tests and compared their performance in a Swedish county. Three of the models outperformed a naïve baseline model where the predictions simply were based on the values observed in the previous week, showing moderate accuracy. In a similar regional context (2), the authors hypothesized that mobile phone mobility patterns are correlated with COVID-19 hospitalizations. The authors made use of the correlation between changes in mobility, resulting in higher SARS-CoV-2 exposure, and presentation of severe clinical COVID-19 symptoms to forecast COVID-19 hospitalizations. They report, from a retrospective case study involving weekly 21-day ahead forecasts provided to two hospitals, that the mean percentage error was below 30% in 16/17 and 8/9 of the forecasts provided.

## Discussion

Our evaluation of forecasting research involving Swedish populations in the vaccination phase of the COVID-19 pandemic identified 6 publications fulfilling evidence quality criteria set by an updated version of ROBOT. Predictive performance was quantified against factual outcomes in some form in five of these publications, but the heterogenicity of methods used disallowed meta-analysis of forecasting performance. In a previous study covering the first pandemic year in Sweden when only NPIs were available, one of five articles reporting forecasts were found to include quantified validation against factual outcomes [17]. A possible explanation for the increased interest in forecasting accuracy is that the present research was reported from a model development perspective, while decision support for policymakers was prioritized in the early pandemic phase.

The usefulness of pandemic forecasts depends on the accuracy of predictions reported, but also on their use value, i.e. whether the predictions are relevant for real-world problem solving. One year into the COVID-19 pandemic, the increased scientific knowledge had allowed development of vaccines for primary prevention of severe illness. Even so, empirical evidence to support health service policymaking was missing in several areas. The first versions of the COVID-19 vaccines were deployed to protect against the ancestral SARS-CoV-2 variant. Although the virus genome had been described, its ecological adaptation patterns were unknown, and consequently, also the vaccine effectiveness against new virus variants of concern resulting from the changes to the genome. There thus was a need to dynamically adapt healthcare resources to shifts in the SARS-CoV-2 variants circulating and the associated variations in vaccine effectiveness. The observed scarcity of forecasting research addressing these issues suggests that while computational forecasting research was performed during the pandemic phase when vaccines were available, the studies did not provide substantial use value to decision-makers in public health and healthcare. The qualitative synthesis of article content showed that the research mainly was deployed in a model design context, where empirical data were analysed only to demonstrate model feasibility. Even though important scientific findings regarding model design were reported, particularly on aggregation of data streams and algorithms, the feasibility demonstrations were hard to translate for use in health service policymaking. For instance, (6) reported a method for nowcasting of true case rates for disease progress indicators associated with reporting delay that showed convincing performance in retrospective analyses of Swedish COVID-19 mortality data. However, mortality is from the policy-making perspective a less useful indicator of pandemic development due to that death occurs downstream in the disease process and cause-of-death data have poor reliability [18]. Data for many upstream indicators of virus transmission, such as positive laboratory test results, are directly recorded into healthcare databases making these indicators less associated with reporting delays and reliability problems. It is also doubtful if the collapsing of outcome variables reported by in (3) is meaningful in a health service context. Each of the included data streams (total incidence, ICU admissions, and mortality) conveys from its temporal position in the disease process unique information for health service planning. Of note, the collaborations between modelers and health service professionals in (1) and (2) reported forecasting endpoints of higher relevance for policymaking, e.g. short-term predictions of positive COVID-19 tests and hospitalization rates.

Considering the model accuracy, no external model validation was reported but five of six articles in the final set included model corroboration data in some form, while estimates of uncertainty in the forecasts were reported in two articles. The low proportion of articles reporting quantification of prediction uncertainty agrees with previous systematic reviews [19]. Even though there are numerous other means of assessing uncertainty, ranging from confidence/credible intervals to sensitivity analysis, these means were not used and systematically reported. These observations are troubling from a methodological viewpoint considering that communication of uncertainty is crucial if computational forecasting is to be used for decision support [20, 21]. Nonetheless, an improvement from previous reviews of computational forecasting during the COVID-19 pandemic [11, 19] was that no mix-ups of the concepts ‘forecast’/’prediction’ and ‘scenario analysis’ were observed in this study [22], which suggests that the terminology explicating the prediction worldview had become clearer, at least for publications dealing with a Swedish context. Moreover, the evaluation of predictions in Sweden during the early pandemic phase showed a dominance of theory-based/mechanistic models, mainly the SEIR (susceptible-exposed-infectious-recovered) compartmental model [17]. In this study, we observed domination of statistical models with only 1/6 articles of adequate quality reporting a compartmental model. A possible explanation is the nowcasting character (short prediction horizons) of the models included in this study. The only article considering a prediction horizon exceeding one month in this study reported use of a mechanistic model (5).

Limitations of our study include the restriction to one single highly developed country. This influences the possibilities to draw generalizable conclusions about computational forecasting during pandemics. A review of computational modeling during the COVID-19 pandemic [23], reported that most studies (69.3%) reported predictions for high-income countries. The role of computational forecasting in the perspective of global COVID-19 pandemic response policymaking has been investigated by Hadley et al. [24]. The authors identified a variety of different modelling infrastructures and proposed recommendations for future practice. Although the study has a global perspective, it focuses primarily on prevention at the national level and does not address the needs of secondary prevention provided through healthcare, which occurs mostly on the regional/local level. To learn more about how computational forecasting can support regional pandemic response policymaking in healthcare, a qualitative analysis of studies covering populations in a larger geographical area, such as the Nordic countries or Europe, could be considered. Such a qualitative assessment would provide a complement to quantitative approaches in that this kind approach may offer insights into *why* forecast or scenarios models succeed or fail to inform policymaking and decision-making. There are several reasons why a qualitative content analysis is useful in this context. First, the small sample does not permit of useful quantitative analyses. Second, a qualitative approach can reveal the underlying reasoning and motivations behind modelling choices that have guided development and implementation of prediction and scenario modelling during COVID-19. This can provide a much richer understanding of how researchers attempt to align models to the (perceived and actual) needs of end-users.

The time frame of this study was chosen in relation to a previous systematic review that investigated the use of computational prediction models in Sweden during 2020. The aim of this study was to investigate how the use of prediction models had evolved during the COVID-19 pandemic in Sweden [11]. Unfortunately, the inclusion and exclusion criteria resulted in only 6 studies (compared to 22 in [11] which only covered one year). While this shows that the interest in prediction models was lower in the later phase of the pandemic it also introduces methodological limitations to our study. The small number of included studies limits the amount of quantitative data we were able to extract in terms of e.g. method of calibration, mention of predictive uncertainty and model validation, and hence weakens the conclusions drawn and the generalisability of our result. However, to counter this drawback we also performed a qualitative analysis of the article content, which yielded important insights concerning the connection to health service decision-making and the primary research aims. While it would not be possible to consider studies published prior to 2021 (since this would overlap with the studies reported in [11]) it is possible that relevant studies were published after 2022. Data from these potential studies is not included in our analysis, which might limit the completeness of the systematic review.

In conclusion, this systematic review of computational forecasting research applied to Swedish populations during the vaccination phase of the COVID-19 pandemic show weak alignment between the research and policymaker needs and a lack of external validation of forecast performance. We can only speculate about the reasons for the poor alignment between research focus and health service needs, but explanations include legislation restricting sharing of sensitive health data with modelers and health service providers being “weak customers” regarding requirements on modeling support. It can also be hypothesized that the vaccination programs and growing natural population immunity in the later pandemic phase increased the complexity of representing relevant health service problems in computational models. Some formal aspects of the scientific reporting of pandemic forecasts had improved compared with the early pandemic phase, such as terminology use, whereas other aspects still displayed room for improvement, e.g. the validation of forecasts against factual outcomes, model documentation, and quantification of uncertainty in the reported predictions. We conclude that more research is warranted on the practical application of computational forecasting in later pandemic phases when representation of relevant health service problems is more challenging.

## Supplementary Information

Below is the link to the electronic supplementary material.

Supplementary Material 1

Supplementary Material 2

## Data Availability

No datasets were generated or analysed during the current study.

## Abbreviations

COVID-19: Coronavirus disease 2019
SARS-CoV-2: Severe acute respiratory syndrome coronavirus 2
NPI / NPIs: Non-pharmaceutical intervention(s)
mRNA: Messenger ribonucleic acid
PROSPERO: International prospective register of systematic reviews
MeSH: Medical Subject Headings
PRISMA-S: Preferred Reporting Items for Systematic reviews and Meta-Analyses — Search extension
PICO: Population, Intervention, Comparison, Outcome
ROBOT: Risk of Bias Opinion Tool
PROBAST: Prediction model Risk Of Bias ASsessment Tool
ICU: Intensive care unit
RMSE: Root mean squared error
MAE: Mean absolute error
CRPS: Continuous ranked probability score
PI: Prediction interval
SEIR: Susceptible–Exposed–Infectious–Recovered
WHO: World Health Organization
