# Supplementary material for: Computational forecasting using Swedish data in the vaccination phase of the COVID-19 pandemic: a systematic literature review deliberating modelling relevance for public health and healthcare
Source: BMC Health Serv Res. 2026 Jul 10;26:947. doi: 10.1186/s12913-026-15076-y (PMC13356788; doi:10.1186/s12913-026-15076-y)
Supplement: Supplementary file 2 — Supplementary Material 2 [file 12913_2026_15076_MOESM2_ESM.docx]

**Supplementary Material 3 - Risk of Bias Opinion Tool (ROBOT)**

**Population data (used for model construction)**

Swedish real-world population (validated/trusted source)

Swedish semi-virtual population concordant with demographic parameters (validated/trusted source)

*CUT-OFF*

Swedish data (validation/source unclear)

No Swedish population data used

Other

Scoring: Below cut-off 2; above cut-off 0

**Time frame of forecast**

< 1 month

1-6 months

7-12 months

*CUT-OFF*

> 12 months

Scoring: Below cut-off 3; above cut-off 0

**Generation of Forecasting model**

Based on current empirical literature (referenced Covid-19 studies)

Knowledge engineering (expert rules)

*CUT-OFF*

Empirical generation (syndromic and diagnostic data / Swedish populations)

Empirical generation (diagnostic data / Swedish populations)

Empirical generation (syndromic data / Swedish populations)

Scoring: Below cut-off 0; above cut-off 1

**Model assumptions (estimates not based on data collected from the Swedish target population)**

Basic reproduction number (*R*o)

Effective reproduction number (*R*)

Latency period

Incubation period

Serial interval

Infectious period

Immunity proportions in population

Case fatality ratio (CFR)

Case hospitalization ratio

Interventions during forecasted period

Scoring: 2 or more assumptions 1; 0-2 assumptions 0

**Basic (Internal/Retrospective) Validation**

No validation

*CUT-OFF 1*

Pure retrospective

*CUT-OFF 2*

Random split

Cross-validation

Bootstrapping

Scoring: Below cut-off 2 0; between cut-off 1-2 1; above cut-off 1 2

**External Validation**

None

*CUT-OFF*

Temporal validation (prospective data from generation site)

Geographical validation (retrospective data from other than generation site)

Fully independent (prospective and geographic)

Scoring: Below cut-off 0; above cut-off 1

**ROBOT score requirement for inclusion in data synthesis (detailed assessment):** ROBOT < 4
